# Case Report: Tissue Expanders—Another Tool in the Armamentarium for the Treatment of Complex Ventral Hernia

**DOI:** 10.3389/jaws.2024.13434

**Published:** 2024-09-20

**Authors:** S. Primrose, M. McClaren, K. Slater

**Affiliations:** Department of Surgery, Princess Alexandra Hospital, Brisbane, QLD, Australia

**Keywords:** tissue expansion, complex abdominal wall reconstruction, component separation, botulinum neurotoxin, giant omphalocele

## Abstract

Complex abdominal wall hernias represent a significant reconstructive challenge to the general surgeon. In patients with loss of abdominal domain, standard surgical techniques such as anterior component separation or transversus abdominus muscle release may not allow for primary fascial closure. In complex ventral wall hernias, visceroabdominal disproportion may need to be addressed prior to an attempt at hernia repair. Tissue expanders placed in the intermuscular space is a novel technique used to increase intraabdominal volume and safely allow reduction of viscera with subsequent closure of the myofascia. We present the case of an adult patient with complications of an untreated congenital omphalocele who underwent a successful two stage operation using tissue expanders in the abdominal wall combined with anterior component separation.

## Introduction

Repair of complex abdominal wall hernias remains a significant surgical challenge for the General Surgeon. In recent years however, improvements in pre-operative management combined with advances in technology and intra-operative techniques have meant that the most complex ventral hernia patients may have the opportunity to have their abdominal wall restored. Patients with loss of domain from lateralisation of abdominal wall musculature or those with large myofascial defects due to previously destroyed tissue usually have a history of extensive abdominal surgery, making this subset of hernias particularly difficult to treat [1]. For these patients, the aims are to restore fascial integrity, provide adequate skin coverage, recovery of functionality and quality of life and prevent hernia recurrence.

For abdominal wall defects that are not amenable to simple tensionless approximation of the natural tissue, various reconstructive methods have been reported. These include preoperative measures such as progressive pneumoperitoneum and injection of botulinum toxin, intraoperative techniques including anterior and posterior component separation techniques and the use of Fasciotens^®^ Hernia [2–7]. Less optimally, techniques to bridge the abdominal wall defect with prosthetic mesh and rotational myocutaneous free flaps have been employed [7, 8]. There are also techniques that require multiple procedures following the index operation, such as mesh mediated fascial traction and proprietary devices such as Fasciotens^®^ Abdomen and the ABRA^®^ device [9, 10].

The use of tissue expansion to create an abdominal domain and lengthen the components of the abdominal wall musculature may also be an effective adjunct [11]. Although commonly used for congenital defects in paediatric patients and to restore lost tissue in adult reconstruction like breast surgery to expand skin to cover defects, the use of tissue expanders in the reconstruction of elective adult abdominal wall hernias remains limited and underutilised [12, 13]. Case reports and case series have described tissue expansion as an effective tool following abdominal compartment syndrome and damage control surgery, however there is no clear technique, indication or consensus on their use [14–16]. We describe a useful and simple application of tissue expanders in abdominal wall reconstruction as described by Jacobsen and colleagues and combined with anterior component separation to successfully manage an adult patient with complications of an untreated congenital omphalocele [5, 11].

## Case Presentation

A 65-year-old lady was referred with a complex abdominal wall defect complicated by an enteroatmospheric fistula in the right iliac fossa. Born with omphalocele in the 1960s, when surgical treatment of this condition was limited, she was cared for by her dedicated mother and a paediatric surgeon who utilised the treatment of the era, escharotic ointment. Eventually, sections of mesh were placed across the viscera. Whilst skin coverage of the visceral block was eventually achieved, at various times in her life, she developed multiple enteroatmospheric fistulae. These were treated by local suture and refeeding enteroclysis. Extraordinarily, there were periods of time where these fistulae healed and the patient progressed to adulthood and has led a prosperous life. Definitive fascial closure of her abdominal wall was never achieved, and she has lived with only native skin and mesh inserted during her childhood protecting the viscera.

**FIGURE 1:**
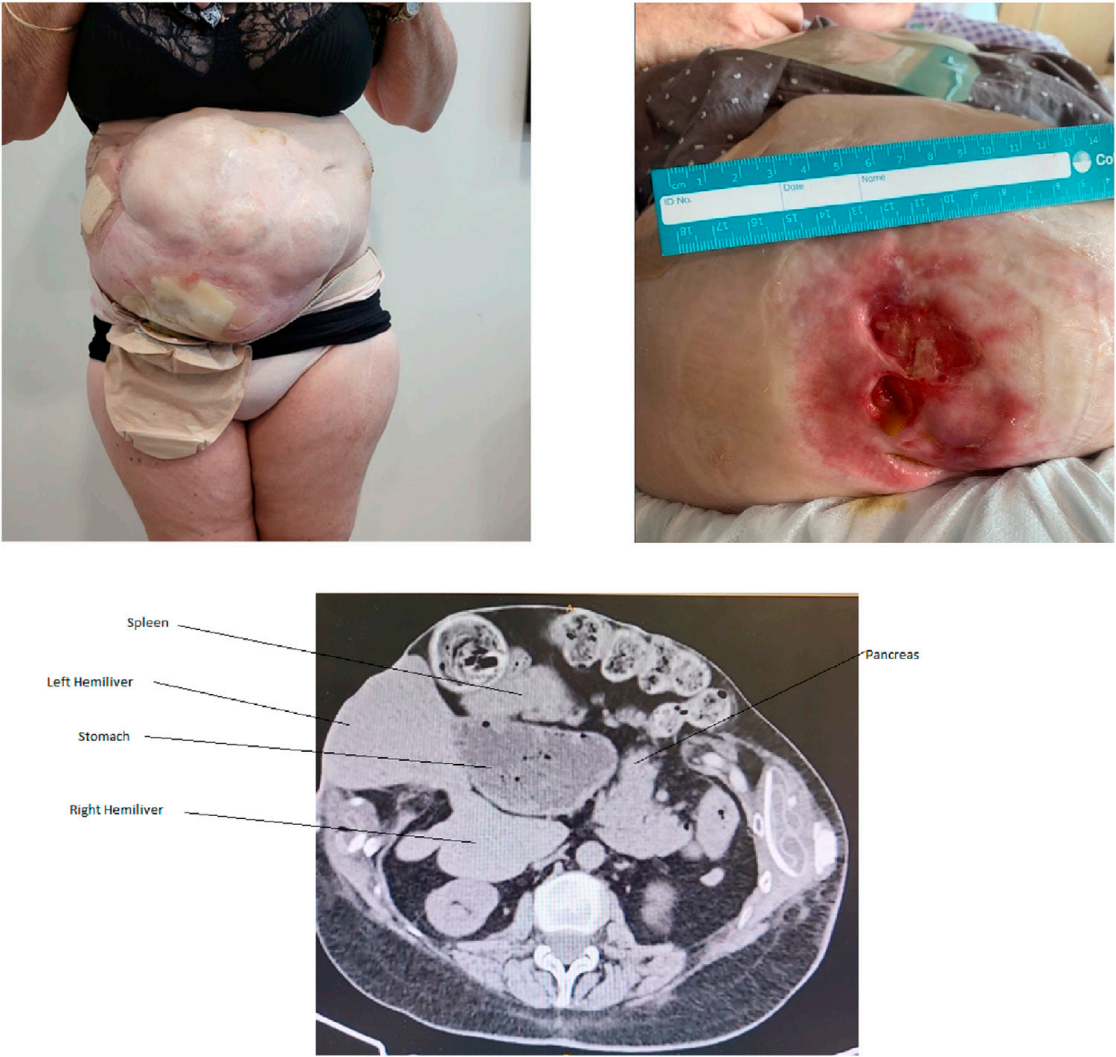
Patient at the time of most recent presentation with high volume output enteroatmospheric fistula involving the lower abdominal wall with severe skin maceration. In addition to 100% loss of domain, CT demonstrates malrotation of the viscera, with the foregut organs occupying a position in the right abdomen.

The patient remained well until the age of 55 when she developed another spontaneous enteroatmospheric fistula which was again treated with local suture of the bowel and wound negative pressure dressing to achieve healing. At age 65, she once again developed a multi-lumen enteroatmospheric fistula in the right iliac fossa. This fistula involved proximal small bowel with an output of approximately 3 L of chyme per day (Figure 1). This was affecting her quality of life due to skin irritation, frequent ostomy changes and she had developed renal impairment due to high fluid losses. Computer Tomography (CT) Scan demonstrated the presence of bilateral rectus abdominis muscles, fascia and all the obliques. These were retracted widely in the flanks consistent with a failure of fusion of the linea alba. The defect was 160 × 209 mm in the transaxial plane. Many of the abdominal organs sat outside of the abdominal cavity and there was significant visceral malrotation (Figure 1). The Tanaka Score was 100% [17]. After several consultations, a plan was made to restore gastrointestinal continuity and attempt abdominal wall advancement toward the midline to prevent future fistulation.

## Surgical Technique

### First Stage Operation: Placement of Expanders

The first stage operation entailed the placement of two tissue expanders between the external and internal oblique muscles. 300 units of botulinum toxin was also injected bilaterally into the external and internal oblique muscles and the transversus abdominis muscles at the same procedure, to facilitate the elongation of the muscles. To place the expanders, bilateral access incisions were made just inferior to the tips of the ninth ribs. Using an open cutdown technique, the external oblique aponeurosis was identified and incised. An inguinal hernia unilateral balloon dissector was inserted inferiorly between internal and external oblique and a 30-degree laparoscope was inserted, to confirm the correct plane. The balloon was then deflated and a Mentor^®^ Smooth 700 millilitre (mL) rectangular tissue expander (8 cm × 15 cm) was placed in the cavity. The process was repeated on the other side. Via an access port, 90 mL of methylene blue was placed in each expander. This serves the purpose of ensuring during subsequent expander fills, that the access needle is in the port (by drawing back on the fluid in the port), rather than a seroma cavity that may surround the port.

### Outpatient Expansion Process

The expander filling process began 1 week after the insertion of the prosthesis. The expanders were filled with 150 mL of sterile saline once a week until they reached their maximum volume. The time between first fill and definitive surgery was 7 weeks. This process was performed as an outpatient in the office. Whilst uncomfortable for the first 24 h, the patient managed with simple analgesia until the last fill, where she required oral narcotics due to the discomfort.

### Second Stage Operation: Removal of Expanders and Anterior Component Separation

**FIGURE 2:**
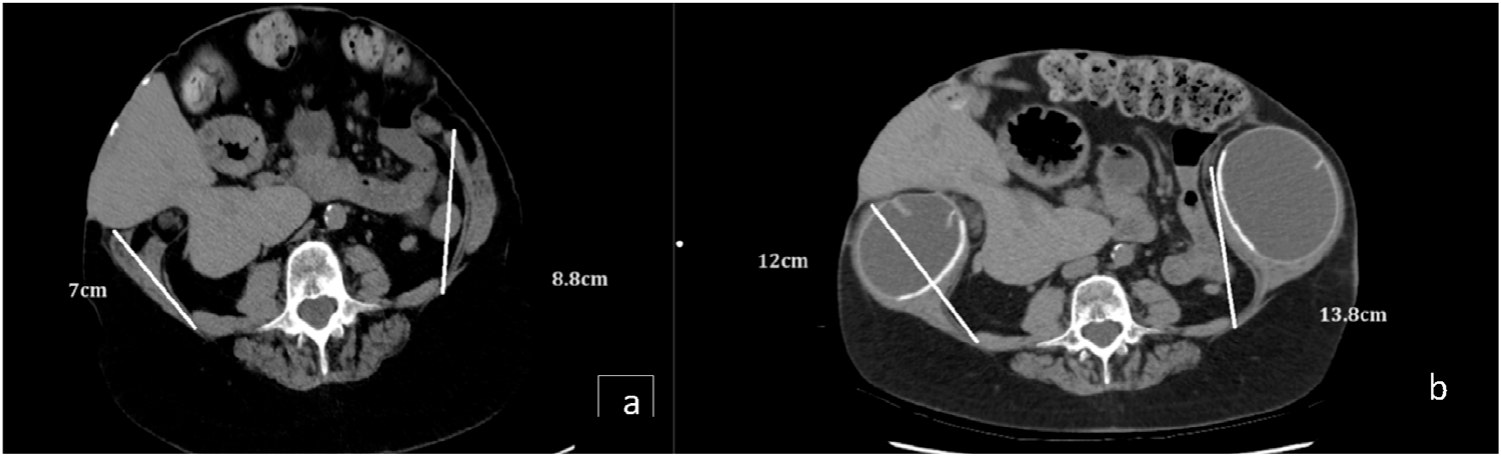
**(A)** Axial CT before expansion axial CT. **(B)** Axial CT demonstrating the additional length gained in the oblique muscles and aponeurosis following full expansion and botox.

The second stage surgery was performed 7 weeks after the commencement of the tissue expansion process. A preoperative CT scan demonstrated 5 centimetres (cm) of elongation of the external oblique muscle and aponeurosis on each side (Figures 2A, B). A midline incision to access the abdominal cavity was used. No formal peritoneum or peritoneal cavity was present and the viscera were matted together. Due to mesh embedded in the skin, there was an eggshell like quality to this tissue and in places the mesh/skin was adherent to the viscera. Once the mesh and skin adherent to the anterior surface of the visceral block were divided, the rest of the bowel loops were straightforward to dissect.

An extended right hemicolectomy was required to repair the jejunal-enteroatmospheric fistula. The capsule of segment III/II of the left hemiliver was densely adherent to the mesh and was very difficult to separate, resulting in severe decapsulation of this section of the liver. Consideration was given to performing a left lateral segment resection, but it was felt that this would detrimentally add to the complexity of the procedure. A drain was placed over this part of the liver in case of post-operative bile leak. The gallbladder was present without attachment to the liver and cholecystectomy was performed to avoid torsion. The spleen and tail of pancreas were in the right upper quadrant and were restored to a more conventional position on the left.

**FIGURE 3:**
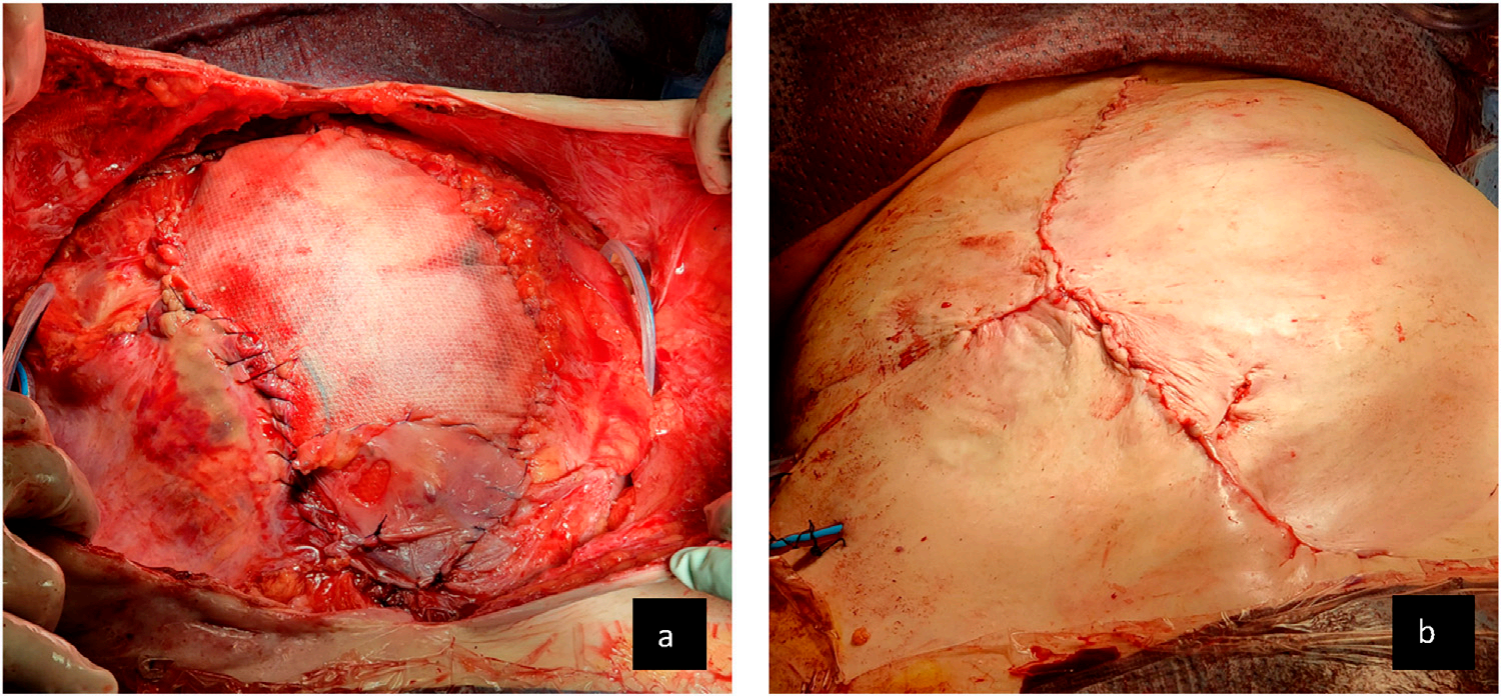
**(A)** Abdominal wall appearance following repair of fistula and external oblique release and medial rotation of external oblique aponeurosis to achieve a bridged closure with wide intraperitoneal mesh. **(B)** Final skin closure over the mesh.

The rectus abdominis muscles and sheaths were separated by 25 cm and were severely atrophic, forming tight, ribbon-like bands in the flanks. There was no possibility of creating a retrorectus plane that would allow their medialisation. Conventional parietal peritoneum was not present and a preperitoneal plane could not be dissected either. The tissue expansion had led to an elongation of the aponeurosis of external oblique and a bilateral external oblique release was performed from over the ribs to just superior to the iliac crest, much more laterally than is usual; approximately 7 cm lateral to the linea semilumaris. The tissue expanders were deflated and removed through the incisions in the external oblique aponeuroses. The elongated fascias created by the expanded external oblique aponeuroses were flipped medially, partially rotating the rectus abdominis muscles. This allowed advancement of native tissue toward the midline, narrowing the defect to approximately 10 cm × 30 cm. Gore^®^ Synecor IP mesh 30 cm × 20 cm was placed intraabdominally, covering the viscera from psoas major muscle to the other. The newly medialised external oblique aponeurosis were sutured around the edge of the mesh with 2.0 PDS (Figure 3A). Excision of the fistula affected skin was performed and the debrided skin was closed over the mesh bridge (Figure 3B). During the surgery, the patient had a period of hypotension, requiring intotropic support. This coincided with manipulation of the defunctioned colon that contained heavily inspissated faeces and led to a systemic immune response process. This settled as the case progressed, the inotropes were weaned, airway pressures were satisfactory and the patient was successfully extubated the following morning.

### Outcome

**FIGURE 4:**
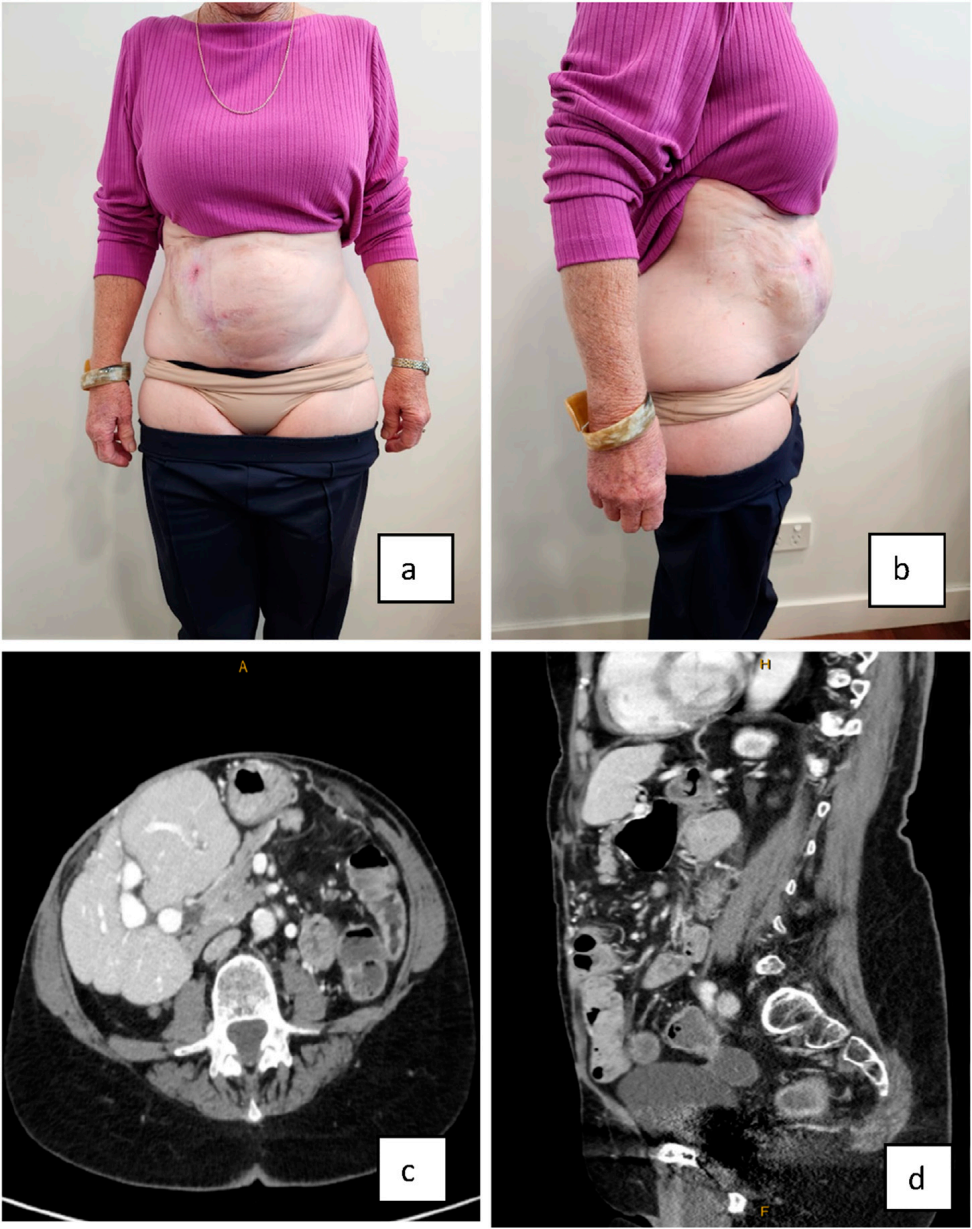
**(A,B)** Final result of abdominal wall closure three and a half years later clinical appearance and imaging. **(C,D)** Axial and sagittal CT scans demonstrating that the loss of domain evident preoperatively, has not recurred and the oblique muscles have remained elongated.

The patient experienced a post operative bile leak from the surface of the liver, where the mobilisation of the old mesh had resulted in hepatic decapsulation. This leak was successfully controlled by the surgical drain and when the bile leak had not resolved after a week, a biliary stent was placed via endoscopic retrograde cholangiopancreatography. There was resolution of the biliary fistula following this. The patient developed a 10 × 2 cm strip of dry necrosis of the skin wound that was left to mummify and contract. At 3.5 years post operatively, there is no recurrent fistula and the patient has retained the integrity of her abdominal wall and her abdominal domain as assessed clinically and radiologically, without significant bulging (Figures 4A–D). The patient reports complete return to all activities without limitation and a vastly improved quality of life. Her kidney function has remained stable.

## Discussion

Patients with abdominal wall failure present the hernia surgeon with multiple challenges, including obesity, medical co-morbidities, gastrointestinal contamination of the abdominal wall, previous surgery, physical and mental deconditioning and loss of abdominal domain. Achieving myofascial closure with stable soft tissue coverage of the viscera is crucial in successful restoration of quality of life to these patients whilst minimising morbidity and mortality. Where there is severe visceroabdominal disproportion, gaining medial advancement of the muscles of the abdominal wall using simple component separation may not be possible without creating compartment syndrome. Achieving this may require multiple, creative and individualised approaches. Hernia surgeons are now fortunate to have many options at our disposal.

Botulinum toxin has proven to be effective at providing preoperative, myofascial elongation which when combined with component separation allows increased midline fascial advancement to facilitate closure [4]. It was an important adjunct in this case to assist in muscles paralysis whilst the tissue expansion occurred. Consideration was given to the use of preoperative progressive pneumoperitoneum, however, given this patient’s abdominal wall had never been medialised and the rigid nature of the skin, it was thought that this technique would not achieve any appreciable improvement in the muscular length [3].

In the time since this case was performed, the Fasciotens ^®^ Abdomen and Fasciotens^®^ Hernia have become available on the Australian market [2]. These devices may have also been valuable intra-operative and post-operative tools, however, given the degree of fibrosis and lateralisation of an abdominal wall that had never before been medialised, gradual pre-operative expansion with the technique described, in addition to botulinum toxin was desired.

Whilst used frequently in plastic surgery, tissue expanders may represent an underutilised tool in the armamentarium of caring for abdominal wall hernias to increase the amount of fascia available for a successful reconstruction [15, 18]. Placement of bilateral tissue expanders in the avascular plane between external and internal obliques allows for the preservation of the neurovascular supply to both the abdominal wall and soft tissue. In addition to the elongation of the external oblique fascia, tissue expanders also increase the area of well vascularised skin available for coverage of the defect, an advantage that turned out to be crucial in this case, as a quarter of this patient’s abdominal skin was unusable due to the presence of a fistula. The use of expanders also has the advantage of permitting a gradual increase in abdominal wall compliance in an outpatient setting titrated to patient comfort. However, use of the tissue expander technique requires good patient compliance with regular expansion and tolerance of the increasing discomfort as the maximal expansion is reached. The optimal timing between maximal expansion and definitive closure is unknown, however this case was limited to 7 weeks due to patient discomfort and requirement of opioid analgesia. Previous authors have allowed anywhere between 6 weeks and 9 months duration for expander treatment [16, 18, 19]. Another disadvantage is the two-stage nature of this approach: the first for tissue expander placement and the second stage for component separation and definitive closure. It may be however, that multiple stage operations may be unavoidable in these complex patients. Expander specific complications including infection, leak and extrusion necessitating immediate removal, however these risks, described by several authors appear to be low [16, 18–20].

Put together, the specific role of tissue expansion in this case was to gradually expand the aponeurosis of the external oblique. Pre-operative paralysis with botulinum toxin was an important adjunct to elongate the muscular components of the obliques. We believe the combination of these two approaches allowed the maximum possible medialisation of the abdominal wall following component separation, minimising the span of bridging mesh required to cover the viscera.

Large studies providing evidence supporting this technique in the reconstruction of complex hernias are not available. This is not surprising given the heterogeneity of this patient cohort, making the design of randomized controlled trials near impossible. Studies are yet to investigate the individual utility of tissue expanders without subsequent component separation as a single technique in the repair of abdominal wall hernia. Despite this, existing case series have reported favourable outcomes in patients undergoing two stage abdominal wall reconstruction using a combined tissue expander and component separation technique. Barron *et al* and Tran *et al* have successfully applied the technique to large incisional hernias following trauma and abdominal compartment syndrome. Barron used tissue expanders in 61 patients, with 56 progressing to hernia surgery utilising anterior component separation. 34% of these patients required bridging mesh and the hernia recurrence rate was 32% [16, 20].

The patient described in this case is rare in that unlike most ventral hernia patients, she is an adult survivor of a congenital abnormality, where her abdominal wall has never been medialised. Due to excellent nursing and medical care, she has lived a fruitful life whilst navigating the evolving techniques of abdominal wall reconstruction. Whilst a unique clinical scenario, this case demonstrates the feasibility of the application of the expander technique to different complex abdominal wall hernias. We believe tissue expansion is relatively simple and is a valuable tool to be considered in select patients in whom single stage component separation or bridging mesh may not achieve adequate tissue coverage of the abdominal viscera. Further studies investigating the utility of tissue expanders both as a combined technique with component separation and individually appear warranted given its successful application in the repair complex abdominal wall hernias.

## Data Availability

The original contributions presented in the study are included in the article/supplementary material, further inquiries can be directed to the corresponding author.

## References

[1] Parker S Mallett L Wood C Boulton R Jamshaid S Erotocritou M Identifying Predictors of Ventral Hernia Recurrence: Systematic Review and Meta-Analysis. Br J Surg Open (2021) 5:6. 10.1093/bjsopen/zraa071 PMC8038271 33839749

[2] Niebuhr H Aufenberg T Dag H Reinpold W Peiper C Schardey HM Intraoperative Fascia Tension as an Alternative to Component Separation. A Prospective Observational Study. Front Surg (2020) 7:616669. 10.3389/fsurg.2020.616669 33708790 PMC7940755

[3] Goni Moreno I. Pneumoperitoneum Applied to the Surgical Preparation of Large Chronic Eventrations. Prensa Med Argent (1971) 58(1):1037–41. 5096685

[4] Elstner KE Read JW Saunders J Cosman PH Rodriguez-Acevedo O Jacombs ASW Selective Muscle Botulinum Toxin A Component Paralysis in Complex Ventral Hernia Repair. Hernia (2020) 24(24):287–93. 10.1007/s10029-019-01939-3 30949893

[5] Ramirez OM Ruas E Dellon AL. “Components Separation” Method for Closure of Abdominal-Wall Defects: An Anatomic and Clinical Study. Plast Reconst Surg (1990) 86(3):519–26. 10.1097/00006534-199009000-00023 2143588

[6] Murr MM Mason EE Scott DH. The Use of Pneumoperitoneum in the Repair of Giant Hernias. Obes Surg (1994) 4(4):323–7. 10.1381/096089294765558278 10742795

[7] Williams KJ Carlson GW deChalain T Howell R Coleman JJ. Role of Tensor Fasciae Latae in Abdominal Wall Reconstruction. Plast Reconst Surg (1998) 101(3):713–8. 10.1097/00006534-199803000-00020 9500388

[8] Nockolds CL Hodde JP Rooney PS. Abdominal Wall Reconstruction With Components Separation and Mesh Reinforcement in Complex Hernia Repair. BMC Surg (2014) 14:25–7. 10.1186/1471-2482-14-25 24886111 PMC4009060

[9] Fung S Ashmawy H Krieglstein C Halama T Schilawa D Fuckert O Vertical Traction Device Prevents Abdominal Wall Retraction and Facilitates Early Primary Fascial Closure of Septic and Non-septic Open Abdomen. Langenbecks Arch Surg (2022) 407(5):2075–83. 10.1007/s00423-021-02424-1 35147749 PMC8832079

[10] Jacobsen WM Petty PM Bite U Johnson CH. Massive Abdominal-Wall Hernia Reconstruction With Expanded External/internal Oblique and Transversalis Musculofascia. Plast Reconst Surg (1997) 100(2):326–35. 10.1097/00006534-199708000-00007 9252598

[11] Clifton MS Heiss KF Keating JJ Mackay G Ricketts RR. Use of Tissue Expanders in the Repair of Complex Abdominal Wall Defects. J Pediatr Surg (2011) 46(2):372–7. 10.1016/j.jpedsurg.2010.11.020 21292090

[12] Martin AE Khan A Kim DS Muratore CS Luks FI. The Use of Intraabdominal Tissue Expanders as a Primary Strategy for Closure of Giant Omphaloceles. J Pediat Surg (2009) 44(1):178–82. 10.1016/j.jpedsurg.2008.10.031 19159740

[13] Celestin AR Barron S Haddad A Jia E Morgenstern M Diamond S Technique of Abdominal Wall Tissue Expansion for the Treatment of Massive Complicated Ventral Hernias. Plast Reconst Surg (2022) 10(2):e4095. 10.1097/GOX.0000000000004095 PMC8830866 35169526

[14] Admire AA Dolich MO Sisley AC Samimi KJ. Massive Ventral Hernias: Role of Tissue Expansion in Abdominal Wall Restoration Following Abdominal Compartment Syndrome. Am Surg (2002) 68(5):491–6. 10.1177/000313480206800520 12013296

[15] Tran NV Petty PM Bite U Clay RP Johnson CH Arnold PG. Tissue Expansion-Assisted Closure of Massive Ventral Hernias. J Am Coll Surg (2003) 196(3):484–8. 10.1016/S1072-7515(02)01896-3 12648704

[16] Ersoy YE Celebi F Erozgen F Ergun SS Akaydin M Kaplan R. Repair of a Post Appendectomy Massive Ventral Hernia Using Tissue Expanders. J Korean Surg Soc (2013) 84(1):61–5. 10.4174/jkss.2013.84.1.61 23323238 PMC3539112

[17] Tanaka E Yoo J Rodrigues A Jr Utiyama E Birolini D Rasslan S. A Computerized Tomography Scan Method for Calculating the Hernia Sac and Abdominal Cavity Volume in Complex Large Incisional Hernia With Loss of Domain. Hernia (2010) 14:63–9. 10.1007/s10029-009-0560-8 19756913

[18] Paletta CE Huang DB Dehghan K Kelly C. The Use of Tissue Expanders in Staged Abdominal Wall Reconstruction. Ann Plast Surg (1999) 42(3):259–65. 10.1097/00000637-199903000-00006 10096616

[19] Barron SL Morgenstern M Jia E Celestin A Diamond S Plaster B The Use of Abdominal Wall Tissue Expansion Prior to Herniorrhaphy in Massive Ventral Hernia Defects. J Plast Reconstr Aesthet Surg (2023) 83:289–97. 10.1016/j.bjps.2023.05.013 37290370

[20] Wang Y Alnumay A Paradis T Beckett A Fata P Khwaja K Management of Open Abdomen After Trauma Laparotomy: A Comparative Analysis of Dynamic Fascial Traction and Negative Pressure Wound Therapy Systems. World J Surg (2019) 43(12):3044–50. 10.1007/s00268-019-05166-w 31506714

